# Changes in insulin receptor signaling underlie neoadjuvant metformin administration in breast cancer: a prospective window of opportunity neoadjuvant study

**DOI:** 10.1186/s13058-015-0540-0

**Published:** 2015-03-03

**Authors:** Ryan JO Dowling, Saroj Niraula, Martin C Chang, Susan J Done, Marguerite Ennis, David R McCready, Wey L Leong, Jaime M Escallon, Michael Reedijk, Pamela J Goodwin, Vuk Stambolic

**Affiliations:** Princess Margaret Cancer Centre, University Health Network, 101 College Street, Toronto Medical Discovery Tower, Room 13-313, Toronto, ON M5G 1L7 Canada; Department of Medical Oncology and Hematology, CancerCare Manitoba and University of Manitoba, Winnipeg, MB Canada; Department of Laboratory Medicine and Pathology, Mount Sinai Hospital, Toronto, ON Canada; Department of Laboratory Medicine and Pathobiology, University of Toronto, Toronto, ON Canada; Campbell Family Institute for Breast Cancer Research and Laboratory Medicine Program, University Health Network, Toronto, ON Canada; Applied statistician, 9227 Kennedy Road, Markham, ON Canada; Department of Surgery, Mount Sinai Hospital, University of Toronto, Toronto, ON Canada; Department of Surgical Oncology, University Health Network, University of Toronto, Toronto, ON Canada; Campbell Family Institute for Breast Cancer Research, Princess Margaret Cancer Centre, Toronto, ON Canada; Division of Medical Oncology and Hematology, Department of Medicine, Mount Sinai Hospital, and Princess Margaret Cancer Centre, University of Toronto, Toronto, ON Canada; Department of Medical Biophysics, University of Toronto, 101 College Street, Toronto Medical Discovery Tower, Toronto, ON M5G 1L7 Canada

## Abstract

**Introduction:**

The antidiabetic drug metformin exhibits potential anticancer properties that are believed to involve both direct (insulin-independent) and indirect (insulin-dependent) actions. Direct effects are linked to activation of AMP-activated protein kinase (AMPK) and an inhibition of mammalian target of rapamycin mTOR signaling, and indirect effects are mediated by reductions in circulating insulin, leading to reduced insulin receptor (IR)-mediated signaling. However, the *in vivo* impact of metformin on cancer cell signaling and the factors governing sensitivity in patients remain unknown.

**Methods:**

We conducted a neoadjuvant, single-arm, “window of opportunity” trial to examine the clinical and biological effects of metformin on patients with breast cancer. Women with untreated breast cancer who did not have diabetes were given 500 mg of metformin three times daily for ≥2 weeks after diagnostic biopsy until surgery. Fasting blood and tumor samples were collected at diagnosis and surgery. Blood glucose and insulin were assayed to assess the physiologic effects of metformin, and immunohistochemical analysis of tumors was used to characterize cellular markers before and after treatment.

**Results:**

Levels of IR expression decreased significantly in tumors (*P* = 0.04), as did the phosphorylation status of protein kinase B (PKB)/Akt (S473), extracellular signal-regulated kinase 1/2 (ERK1/2, T202/Y204), AMPK (T172) and acetyl coenzyme A carboxylase (S79) (*P* = 0.0001, *P* < 0.0001, *P* < 0.005 and *P* = 0.02, respectively). All tumors expressed organic cation transporter 1, with 90% (35 of 39) exhibiting an Allred score of 5 or higher.

**Conclusions:**

Reduced PKB/Akt and ERK1/2 phosphorylation, coupled with decreased insulin and IR levels, suggest insulin-dependent effects are important in the clinical setting. These results are consistent with beneficial anticancer effects of metformin and highlight key factors involved in sensitivity, which could be used to identify patients with breast cancer who may be responsive to metformin-based therapies.

**Trial registration:**

ClinicalTrials.gov identifier: NCT00897884. Registered 8 May 2009.

**Electronic supplementary material:**

The online version of this article (doi:10.1186/s13058-015-0540-0) contains supplementary material, which is available to authorized users.

## Introduction

The antidiabetic drug metformin has emerged as a potential anticancer agent. Metformin is commonly used to treat type 2 diabetes because of its ability to reduce circulating glucose and insulin levels. Researchers in several retrospective studies have reported that patients with diabetes receiving metformin exhibited decreased cancer incidence and cancer-related mortality [1-3]. Metformin use has also been associated with increased pathologic complete response rates to neoadjuvant therapy [4]. However, these retrospective studies are subject to selection and time-related biases, highlighting the need for well-designed prospective clinical trials [5]. Metformin also has direct growth-inhibitory effects on breast cancer cells in culture and reduces mammary tumor growth in mice [6-8]. The proposed mechanism(s) of antitumor action of metformin involves both direct (insulin-independent) and indirect (insulin-dependent) actions of the drug. The indirect, insulin-dependent effects of metformin are associated with reductions in circulating insulin levels in patients [9,10] and are likely mediated by inhibition of gluconeogenesis in the liver and increased glucose uptake in muscle [11,12]. Given the mitogenic and antiapoptotic effects of insulin, the reduction in systemic levels may be critical to the anticancer effects of metformin, particularly in cancers associated with obesity and hyperinsulinemia, such as those of the breast and colon [13-16]. Moreover, certain cancers, including breast, express high levels of the insulin receptor (IR), and increased circulating insulin is associated with breast cancer recurrence and death [13,17,18]. Thus, metformin may diminish the stimulatory effects of insulin on breast cancer.

**Figure 1:**
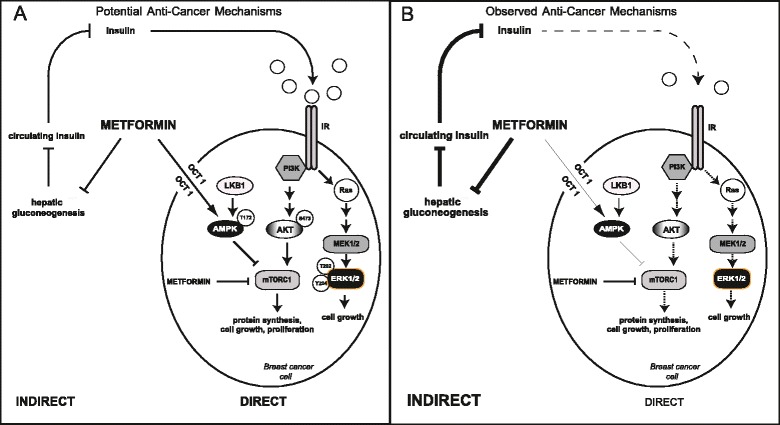
**Proposed mechanism of action of metformin. (A)** The potential mechanism(s) of antitumor action of metformin involves both direct (insulin-independent) and indirect (insulin-dependent) actions of the drug. The direct, insulin-independent effects of metformin involve activation of AMP-activated protein kinase (AMPK) in cancer cells via phosphorylation on Thr172 by liver kinase B1 (LKB1) and a subsequent reduction in mammalian target of rapamycin (mTOR) signaling, protein synthesis and cell proliferation. AMPK-independent actions of metformin may also contribute to its anticancer effects. The indirect, insulin-dependent effects are associated with reductions in circulating insulin levels mediated by inhibition of hepatic gluconeogenesis. The resulting decrease in insulin leads to reduced insulin receptor (IR)-mediated cancer cell signaling. **(B)** The results of the present study indicate that insulin-dependent effects of metformin are important in the clinical setting. Decreases in circulating insulin levels in metformin-treated patients, coupled with reductions in Akt (S473) and extracellular signal-regulated kinase 1/2 (ERK1/2; T202/Y204) phosphorylation in breast cancer cells, suggest reduced IR activation followed by decreases in phosphatidylinositol 3-kinase (PI3K) and Ras-mitogen-activated protein kinase (MAPK) signaling, cell proliferation and survival. MEK1/2, Mitogen-activated protein kinase kinase 1/2; mTORC1, Mammalian target of rapamycin complex 1; OCT1, Organic cation transporter 1.

The direct effects of metformin have been attributed to the activation of AMP-activated protein kinase (AMPK), a serine/threonine protein kinase involved in maintaining metabolic homeostasis that is sensitive to increases in the intracellular ratio of AMP to ATP [19,20]. AMPK acts as a cellular energy sensor and phosphorylates a number of effectors involved in the stimulation of ATP-generating pathways and the inhibition of ATP-consuming pathways [19]. Metformin activates AMPK indirectly by inhibiting mitochondrial respiration and causing a rise in the cellular levels of AMP [21]. The direct anticancer effects of metformin involve activation of AMPK via phosphorylation on Thr172 by the tumor suppressor liver kinase B1 (LKB1) and a subsequent reduction in mammalian target of rapamycin (mTOR) signaling, protein synthesis and cell proliferation (Figure 1A) [22]. Moreover, AMPK-independent direct effects of metformin may contribute to metformin’s mechanism of action [23].

Although the results of numerous epidemiological and preclinical studies support a beneficial effect of metformin in breast cancer, the impact of metformin on cancer in patients who do not have diabetes remains unknown. We recently conducted a neoadjuvant, single-arm, “window of opportunity” trial that characterized the clinical and biological effects of metformin in patients with locoregional breast cancer who did not have diabetes. The clinical effects of metformin have been reported previously [24] and include significant decreases in body mass index (BMI), weight, glucose and homeostatic model assessment (HOMA; an estimate of insulin resistance [25]). A decrease in insulin levels approached significance and was likely due to the small sample size (n = 39) and short duration of metformin treatment. Significant decreases in tumor cell proliferation and increases in apoptosis were also observed after metformin treatment [24]. To explore the molecular changes associated with the systemic effects of metformin and to elucidate its mechanism of antitumor action, additional immunohistochemical analyses focused on receptor expression and intracellular signaling in tumor tissue were performed. In this article, we present our results.

## Methods

### Clinical study

A single-arm, “window of opportunity” neoadjuvant metformin study (ClinicalTrials.gov identifier: NCT00897884) was conducted at Mount Sinai and Princess Margaret hospitals in Toronto, ON, Canada, from March 2009 to June 2011 (Additional file 1) [24]. Women with newly diagnosed, treatment-naive, early-stage breast cancer were recruited for participation in the study, regardless of tumor subtype. Patients <70 years of age with normal hepatic, cardiac and renal function were eligible, whereas those receiving neoadjuvant therapy, with known diabetes, blood glucose of ≥7 mmol/L or exposure to metformin in the preceding 4 weeks were ineligible. Eligible patients were administered 500 mg of metformin three times daily for ≥2 weeks after diagnostic core biopsy up to and including the night before surgery. Diagnostic core biopsy and surgical tumor specimens were collected, fixed in formalin and embedded in paraffin (FFPE) pre- and post-metformin treatment, respectively. Core biopsy samples were placed immediately in formalin and formalin-fixed for 8 to 24 hours. Excisional samples were fixed in formalin for a minimum of 24 hours and a maximum of 96 hours, with most fixed for under 72 hours. Tumor characteristics were obtained from standardized pathology reports. All patients included in the study provided informed consent for participation in the trial and for the publication of associated results. The study was conducted in compliance with the research ethics requirements of Princess Margaret and Mount Sinai hospitals and was approved by the Ontario Cancer Research Ethics Board.

### Optimization and validation of antibodies for immunohistochemistry

To ensure optimal performance and to confirm specificity, antibodies against phosphorylated AMPK (p-AMPK) T172, phosphorylated acetyl coenzyme A carboxylase (p-ACC) S79, phosphorylated protein kinase B (p-PKB)/Akt S473 and phosphorylated extracellular signal-regulated kinase (p-ERK) T202/Y204 were optimized for immunohistochemistry (IHC) using FFPE MCF-7 breast cancer cells (American Type Culture Collection, Manassas, VA, USA). For p-PKB/Akt S473 antibody optimization, MCF-7 cells were starved of serum overnight and then treated with either 200 nM of the dual phosphatidylinositol 3-kinase/mammalian target of rapamycin (PI3K/mTOR) inhibitor BEZ235 (Selleck Chemicals, Houston, TX, USA) for 1 hour or media containing 10% fetal bovine serum (Gibco/Life Technologies, Burlington, ON, Canada) and 100 ng/ml heregulin (R&D Systems, Minneapolis, MN, USA) for 20 minutes. To optimize the antibody against p-ERK1/2 T202/Y204, MCF-7 cells were starved of serum overnight and then treated with either 10 μM of the Mitogen-activated protein kinase kinase 1/2 inhibitor U0126 (Sigma-Aldrich, St Louis, MO, USA) for 1 hour or media containing 100 ng/ml epidermal growth factor (EGF; PeproTech, Rocky Hill, NJ, USA) for 10 minutes. For p-AMPK T172 and p-ACC S79 antibodies, MCF-7 cells were either left alone or treated with 2 mM 5-aminoimidazole-4-carboxamide-1-β-d-ribofuranoside (AICAR; Toronto Research Chemicals, Toronto, ON, Canada) for 3 hours. After each respective treatment, cells were collected on ice and divided into two aliquots: one for lysis and Western blot analysis and the other for fixing, embedding and IHC analysis. Immunohistochemical staining (performed as described below) for p-PKB/Akt S473 was observed in serum- and heregulin-stimulated cells as both nuclear and cytoplasmic, whereas treatment with BEZ235 significantly reduced S473 staining (Additional file 2). Staining for p-ERK1/2 T202/Y204 was extremely low in U0126-treated cells, but it was increased in cells treated with EGF (Additional file 2). Likewise, staining for p-AMPK T172 and p-ACC S79 was evident in AICAR-treated cell pellets, and signaling for these proteins was diminished in untreated cells (Additional file 3). The signaling changes observed by IHC were validated for each antibody by Western blot analysis. Additional antibody optimization for IHC was also performed using tissue sections from FFPE human breast tumors to ensure an adequate dynamic range of staining in standard pathology samples.

The antibody for organic cation transporter 1 (OCT1) was optimized and validated using tissue sections from normal human liver and breast, as well as breast tumor tissue (Additional file 4). The IR antibody has been described previously [17].

### Immunohistochemistry

Staining for p-AMPK T172 (1:50 dilution; Cell Signaling Technology, Danvers, MA, USA), p-ACC S79 (1:100 dilution; EMD Millipore, Billerica, MA, USA), p-ERK1/2 T202/Y204 (1:50 dilution; Cell Signaling Technology) and OCT1 (1:75 dilution; LifeSpan BioSciences, Seattle, WA, USA) was performed on a Ventana BenchMark XT automated slide preparation system using the iVIEW 3,3′- diaminobenzidine (DAB) detection kit (both from Ventana Medical Systems, Tucson, AZ, USA) and the IR (1:50 dilution; EMD Millipore) was assessed using the ultraView Universal DAB detection kit (Ventana Medical Systems). For p-PKB/Akt S473, staining was performed manually. Sections were incubated overnight in primary antibody (1:50 dilution; Cell Signaling Technology), and biotin-streptavidin-horseradish peroxidase secondary antibodies (Vector Laboratories, Burlingame, CA, USA; and ID Labs, London, ON, Canada) and DAB were used for detection. Sections from embedded MCF-7 cell pellets (described above) were included as controls for each batch of tumor sections.

All sections were scored manually by a pathologist (MCC) using the Allred system [26], which incorporates the proportion of cells staining positive (scored from 0 to 5) and intensity of staining (scored from 0 to 3) to yield values of 0 or 2 to 8. Scoring was performed in a blinded fashion; the pathologist was unaware of which biopsy and surgical specimens were from the same patient. For p-PKB/Akt, scores were generated for both nuclear and cytoplasmic staining, and an overall score was calculated as the mean of both values.

### Statistical analysis

Because many of the variables had markedly skewed distributions, nonparametric rank-based methods were used to analyze the data, and the medians (upper limit of first and third quartiles (interquartile range, IQR)) were used to summarize the distributions. Change in biomarker scores was calculated as postmetformin minus premetformin, and the Wilcoxon signed-rank test was used to test whether the change was distributed symmetrically around zero. To investigate the extent to which changes in serum insulin, tumor IR expression and p-Akt within the same patient correlated with the patient’s change in Ki67, we scaled the insulin, IR and p-Akt variables such that each one’s largest observed reduction became −1. The three variables were then summed, resulting in a summary variable with the properties that a person with large reductions in all three variables would score near −3 and one with little change in any of the three would score near zero. Spearman’s rank correlations were used to quantify the correlations between variables.

## Results

### Patient cohort and tumor characteristics

The patient cohort has been described previously [24]. Forty-eight patients gave consent and were enrolled in the trial. Nine patients did not complete the trial: three because of side effects, two who were found to have metastatic lesions and became ineligible, two who had surgery dates rescheduled and became ineligible, one who refused the baseline interview and one who had surgery at a nonparticipating hospital. Thirty-nine patients with a mean age of 51 years completed the study, receiving metformin for a median of 18 days (range: 13 to 40). The majority of tumors were of low or intermediate grade and stage (59% grade II or lower, 92% stage II or lower) and 62% (24 of 39) were node-negative (Additional file 5). Eighty-five percent were hormone receptor–positive (33 of 39 estrogen receptor–positive (ER+), 32 of 39 progesterone receptor–positive (PR+)). Five tumors exhibited human epidermal growth factor receptor 2 (Her2) expression, and three were triple-negative (ER−, PR−, Her2−).

### Characterization of indirect effects of metformin

**Figure 2:**
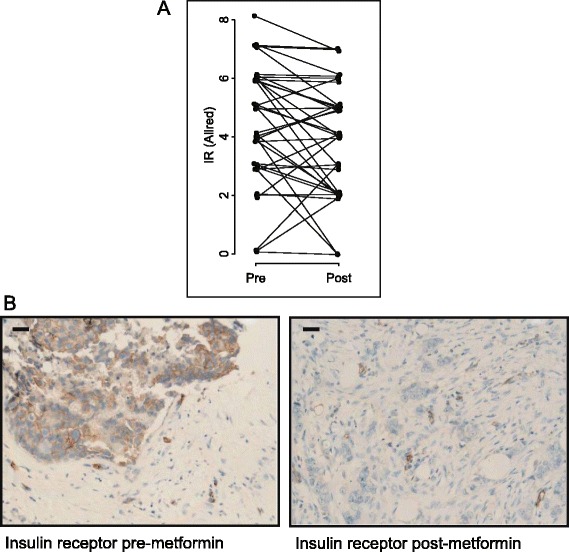
**Changes in insulin receptor expression upon metformin treatment. (A)** Allred scores for the insulin receptor (IR) as measured pre- and post-metformin treatment. **(B)** Representative images are shown for IR pre- and post-metformin treatment with Allred scores of 6 and 2, respectively. Scale bar represents 30 μm.

**Figure 3:**
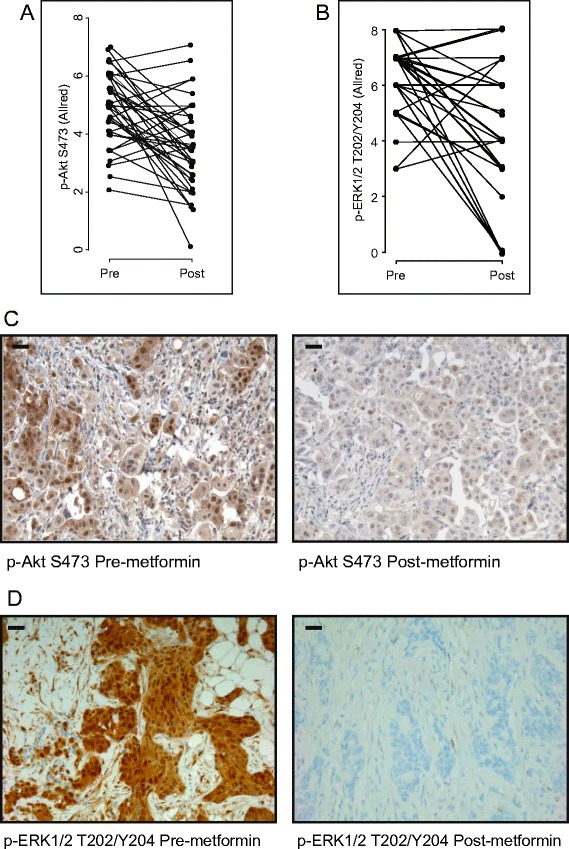
**Changes in phosphorylated Akt and phosphorylated extracellular signal-regulated kinase 1/2 levels upon metformin treatment.** Allred scores for **(A)** phosphorylated Akt (p-Akt) S473 and **(B)** phosphorylated extracellular signal-regulated kinase 1/2 (p-ERK1/2) T202/Y204 as measured pre- and post-metformin treatment. The overall score (average of nuclear and cytoplasmic scores) for p-Akt is shown. **(C)** and **(D)** Immunohistochemical staining of tumor tissue for p-Akt S473 **(C)** and p-ERK1/2 T202/Y204 **(D)**. Representative images are shown for each phospho-protein pre- and post-metformin treatment. Corresponding Allred scores were as follows: p-Akt S473 pre-metformin = 7, post-metformin = 3; p-ERK1/2 T202/Y204 pre-metformin = 8, post-metformin = 0. Scale bar represents 30 μm.

To evaluate the indirect effects of metformin, diagnostic core biopsy and surgical specimens were assessed for expression of the IR and activation of PI3K and Ras-mitogen-activated protein kinase (MAPK) signaling by IHC. Consistent with previous reports [17,27], almost all tumors expressed some level of the IR (only three were negative) at baseline, with a median Allred score of 4.5 (IQR: 3 to 6) (Table 1). Following metformin treatment, levels of IR expression exhibited a median score of 4 (IQR: 2 to 5) (Figure 2A and 2B). The IR, as well as the hybrid IR/insulin-like growth factor 1 receptor (IGFR-1), lie upstream of various mitogenic and prosurvival signaling pathways, including the PI3K/PKB/Akt/mTOR and Ras-MAPK signaling networks [28,29]. The phosphorylation of PKB/Akt (Ser473) was assessed in tumor tissue as a marker of PI3K pathway activation and potential insulin-dependent effects of metformin. Consistent with reduced PI3K signaling, the overall phosphorylation of PKB/Akt decreased from a median score of 5 (IQR: 4 to 6) at baseline to 3.5 (IQR: 2.5 to 4.5) (*P* = 0.0001) after metformin treatment (Figure 3A and 3C). The levels of p-PKB/Akt in the cytoplasm decreased from a median of 6 (IQR: 5.5 to 7) to 3 (IQR: 3 to 5) (*P* < 0.0001) (Additional file 6), whereas nuclear p-PKB/Akt was reduced from a median of 4 (IQR: 2 to 5.5) to 3 (IQR: 2 to 4.5) (*P* = 0.21) (Additional file 6). To evaluate Ras-MAPK signaling in tumors, the phosphorylation of ERK1/2 (T202/Y204) was assessed. The phosphorylation of ERK1/2 (T202/Y204) decreased from a median score of 7 (IQR: 6 to 7) at baseline to 4 (IQR: 3 to 6) (*P* < 0.0001) after metformin treatment (Figure 3B and 3D).

**Table 1:** **Changes in protein levels and phosphorylation pre- and post-metformin** ^**a**^

**Variable**	**Premetformin**	**Postmetformin**	**Change**	***P***-**value**
	**Median (Q1, Q3)**	**Median (Q1, Q3)**	**Median (Q1, Q3)**	
Insulin receptor	4.5 (3, 6)	4 (2, 5)	0 (−1, 0)	0.0375
OCT1	ND	7 (6, 7)	ND	ND
Cytoplasmic p-Akt (S473)	6 (5.5, 7)	3 (3, 5)	−2 (−3, −1)	<0.0001
Nuclear p-Akt (S473)	4 (2, 5.5)	3 (2, 4.5)	0 (−2, 1)	0.206
Overall p-Akt (S473)	5 (4, 6)	3.5 (2.5, 4.5)	−1.5 (−2.5, −0.2)	0.0001
p-ERK (T202/Y204)	7 (6, 7)	4 (3, 6)	−2 (−4, 0)	<0.0001
p-AMPK (T172)	7 (5.2, 7)	5 (4, 6)	−1.5 (−3, 0)	0.0034
p-ACC (S79)	5 (4, 6)	4 (3, 5)	−1 (−2, 1)	0.0193

^a^ND, Not determined; OCT1, Organic cation transporter 1; p-ACC, Phosphorylated acetyl coenzyme A carboxylase; p-AMPK, Phosphorylated AMP-activated protein kinase; p-ERK, Phosphorylated extracellular signal-regulated kinase. The median, first quartile (Q1) and third quartile (Q3) data are listed. Allred scoring was used for each protein. OCT1 expression was examined only in the post-metformin treatment surgical specimens. The *P*-values are derived from Wilcoxon signed-rank tests, which test whether the change scores are distributed symmetrically around zero. The median change for the insulin receptor (IR) registered as zero because of the discrete nature of the data, but the change in IR expression was not symmetric around zero (*P* = 0.04 by Wilcoxon signed-rank test), with 18 of 38 tumors registering a reduction versus 8 that increased (no change in 12, and 1 unevaluable).

### Characterization of direct effects of metformin

**Figure 4:**
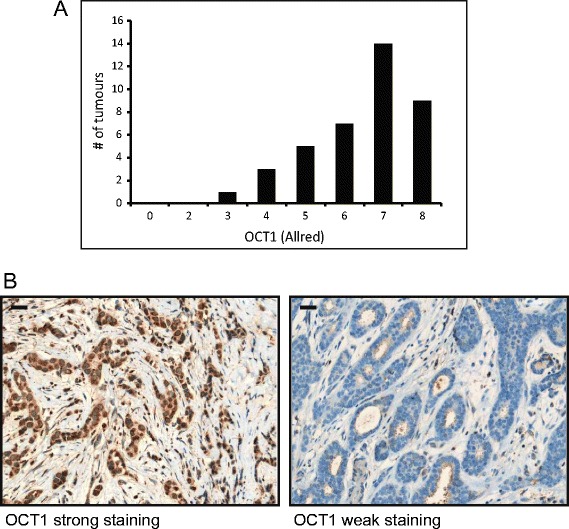
**Organic cation transporter 1 expression in breast tumors.** Allred scores for organic cation transporter 1 (OCT1) as measured in the surgical tumor specimen (post-metformin). **(A)** Graph depicting distribution of OCT1 Allred scores. **(B)** Immunohistochemical staining of tumor tissue for OCT1. Representative images are shown for strong (Allred 8) and weak (Allred 3) staining. Scale bar represents 30 μm.

To determine the extent of the direct effects of metformin, surgical tumor sections were assessed by IHC for expression of OCT1, the transporter required for cellular uptake of metformin [30]. All tumors expressed some level of OCT1, with most (35 of 39) exhibiting an Allred score of 5 or higher (Figure 4A and 4B), indicating the potential for metformin uptake by tumor cells and sensitivity to the direct effects of the drug.

**Figure 5:**
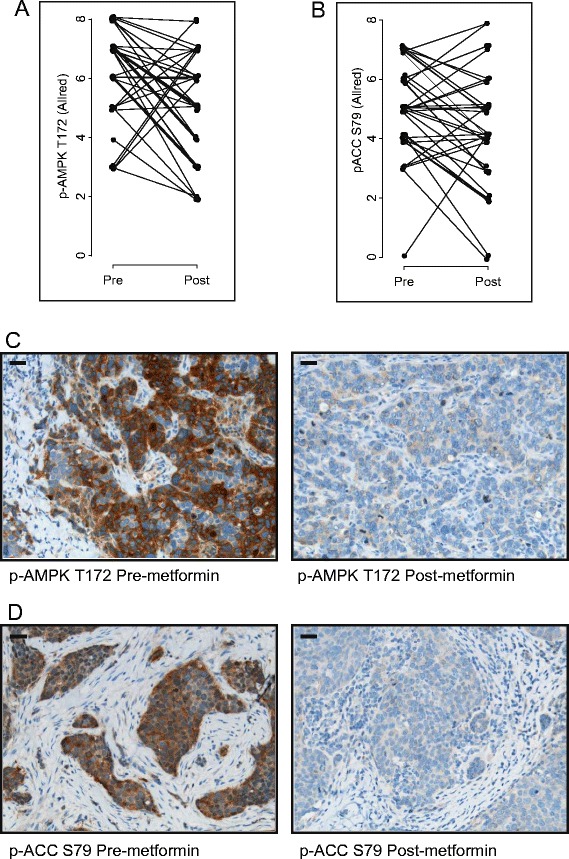
**Changes in phosphorylated AMP-activated protein kinase and phosphorylated acetyl coenzyme A carboxylase levels upon metformin treatment.** Allred scores for **(A)** phosphorylated AMP-activated protein kinase (p-AMPK) T172 and **(B)** phosphorylated acetyl coenzyme A carboxylase (p-ACC) S79 as measured pre- and post-metformin treatment. Immunohistochemical staining of tumor tissue for **(C)** p-AMPK T172 and **(D)** p-ACC S79. Representative images are shown for each phospho-protein pre- and post-metformin treatment. Corresponding Allred scores were as follows: p-AMPK T172 pre-metformin = 8, post-metformin = 4; p-ACC S79 pre-metformin = 7, post-metformin = 4. Scale bar represents 30 μm.

In preclinical models, many of the direct anticancer effects of metformin have been attributed to activation of AMPK via increased T172 phosphorylation by LKB1 [22]. The phosphorylation of AMPK (T172) was assessed at baseline and post-metformin treatment by IHC. AMPK phosphorylation was high at baseline, with a median Allred score of 7 (IQR: 5.2 to 7) (Table 1, Figure 5A and 5C). Following metformin treatment, p-AMPK levels decreased to a median of 5 (IQR: 4 to 6) (*P* < 0.005). The high baseline level of p-AMPK was in contrast to a report of low AMPK phosphorylation in breast tumors [31]. We therefore further explored the activation status of AMPK by measuring the phosphorylation of ACC (Ser79), its direct substrate. Consistent with the apparent AMPK activation in tumors, the phosphorylation of ACC (S79) was high at baseline and decreased following metformin treatment from a median of 5 (IQR: 4 to 6) to 4 (IQR: 3 to 5) (*P* = 0.02) (Figure 5B and 5D). The levels of AMPK and ACC phosphorylation correlated pre- and post-metformin treatment (respectively: Spearman’s rank correlation (*r*) = 0.32, *P* = 0.05; *r* = 0.42, *P* = 0.01), as did the change in phosphorylation of both proteins (*r* = 0.35, *P* = 0.04) (Additional file 7).

### Correlative analyses and assessment of predictors of change in cell signaling

To investigate potential predictive biomarkers of metformin benefit in the neoadjuvant breast cancer setting, a series of correlative analyses with patient and tumor characteristics were conducted (Additional file 7). Consistent with the possibility of insulin stimulating PI3K signaling in tumors, higher overall baseline phosphorylation of PKB/Akt was associated with greater baseline insulin levels (*r* = 0.34, *P* = 0.04). Furthermore, baseline phosphorylation of cytoplasmic PKB/Akt was higher in tumors with greater baseline proliferation levels as measured by Ki67 staining (*r* = 0.41, *P* = 0.01), and significantly greater reductions in Ki67 were seen in tumors with larger decreases in overall PKB/Akt phosphorylation (*r* = 0.37, *P* = 0.0225) (Additional file 7). Whereas baseline insulin levels were associated with increased PKB/Akt signaling and the latter with tumor cell proliferation, changes in PKB/Akt phosphorylation were not associated with changes in circulating insulin levels (*r* = 0.02, *P* = 0.90). However, when patients were considered individually, those exhibiting the largest reductions in serum insulin, tumor IR expression and p-Akt (as a summary variable) exhibited the largest reductions in tumor cell proliferation (*r* = 0.41, *P* = 0.012) (Additional file 8).

## Discussion

Although preclinical and epidemiological data support the use of metformin for the treatment of breast cancer, well-designed prospective clinical trials are necessary to evaluate the biological effects of the drug in patients without diabetes. In the present study, women with early stage breast cancer who did not have diabetes were treated with metformin in the neoadjuvant setting, and immunohistochemical analysis of tumor material was used to investigate its effects on tumor cell signaling and evaluate potential direct and indirect mechanisms of action. Metformin had a significant impact on signaling in tumor cells, as expression of the IR and phosphorylation of PKB/Akt and ERK1/2 decreased upon treatment. All tumors expressed OCT1, indicating the potential for metformin uptake by breast cancer cells. However, surprisingly, metformin induced decreases in the phosphorylation of AMPK and ACC in tumor tissue.

Insulin stimulates breast cancer cell growth and survival *in vitro*, and hyperinsulinemia is an adverse prognostic factor in breast cancer [13,29]. As previously reported [27,17], the majority of tumors (36 of 39) expressed the IR, indicating their potential sensitivity to mitogenic effects of insulin and its decrease following exposure to metformin. Interestingly, IR expression significantly decreased upon metformin treatment, possibly in relation to the decrease in circulating insulin induced by this drug. Previously, a reduction in tumor ER expression was reported in patients with breast cancer treated with the aromatase inhibitor exemestane [32], indicative of a relationship between ligand and receptor levels.

The IR lies upstream of several mitogenic pathways implicated in cancer, including the PI3K/PKB/Akt/mTOR and Ras-MAPK signaling networks [28,29]. Moreover, IGFR-1, also proposed to be involved in cancer, can hybridize with the IR to bind insulin and stimulate cell proliferation [29]. IR activation and insulin-lowering effects of metformin were evaluated in tumors by assessing the phosphorylation of downstream effectors of PI3K (PKB/Akt S473) and Ras-MAPK (ERK1/2 T202/Y204) at baseline and post-metformin treatment. At baseline, the overall phosphorylation of PKB/Akt correlated with circulating insulin levels, and phosphorylation (overall and cytoplasmic) decreased significantly upon metformin treatment. Likewise, the phosphorylation of ERK1/2 decreased in tumors after administration of metformin. Expression of the IR, coupled with the decrease in PKB/Akt and ERK1/2 activity in metformin-treated tumors, supports a model whereby lowering of systemic insulin leads to decreased IR stimulation on tumor cells and a subsequent reduction in downstream signaling, cell proliferation and survival (Figure 1B). Indeed, a 10% decrease in circulating insulin levels was observed [24], and greater reductions in cell proliferation (as measured by Ki67 staining [24]) were seen in tumors with the greatest decreases in overall PKB/Akt phosphorylation (*r* = 0.37, *P* = 0.02). These results parallel those obtained in mouse models, where metformin, while failing to activate AMPK in tumors, reduced systemic insulin levels and decreased PI3K/PKB/Akt/mTOR and ERK1/2 signaling in tissue [33]. Furthermore, when serum insulin levels, tumor IR expression and Akt phosphorylation were grouped as a summary variable and patients were assessed individually, those exhibiting the largest reductions in insulin, tumor IR and p-Akt exhibited the largest reductions in tumor cell proliferation (Additional file 8) (*r* = 0.41, *P* = 0.012), supporting a role for the insulin-dependent effects of metformin in its mechanism of antitumor action. However, overall changes in PKB/Akt and ERK1/2 phosphorylation did not correlate with reductions in circulating insulin levels (respectively: *r* = 0.02, p = 0.90; *r* = −0.09, p = 0.61) (Additional file 7), warranting additional work aimed at understanding the extent of the indirect effects of the drug.

The direct effects of metformin *in vitro* are associated with AMPK activation and inhibition of mTOR signaling [22]. A critical and rate-limiting step in metformin-mediated AMPK activation is its cellular uptake. Metformin is transported across cell membranes by OCT1, OCT2 and OCT3. The OCT transporters belong to the solute carrier 22 family of transport proteins, and genetic polymorphisms in the gene encoding OCT1 are known to affect the sensitivity of patients to metformin [30,34]. In addition, deletion of OCT1 in mice leads to reduced hepatic accumulation of metformin, a reduction in metformin-mediated AMPK and ACC phosphorylation, and resistance to the glucose-lowering effects of the drug [30]. Although OCT1 is found in normal mammary epithelial cells, its expression in breast tumors is not known [35,36]. Immunohistochemical analysis of specimens revealed OCT1 expression in every breast tumor (n = 39), with the majority exhibiting an Allred score of 5 or higher. The presence of OCT1 formally supports the possibility of tumor sensitivity to the direct effects of metformin mediated by AMPK activation. Nevertheless, AMPK activity in tumors, as assessed by T172 phosphorylation, was already high at baseline and decreased upon metformin treatment (Figure 5A). The high level of AMPK phosphorylation found in untreated breast tumors is in contrast to a previous report of limited AMPK activation in breast cancers [31]. However, the level of AMPK activation observed in the present study was corroborated by staining of tumors for the phosphorylation status of the AMPK substrate ACC (S79) (Figure 5B). The discrepancy in the level of AMPK phosphorylation may be a result of technical differences in tissue extraction, fixation and antigen retrieval or the use of tumor biopsies (present study) versus tissue microarrays [31]. Further complicating the assessment is the unexpected decrease in AMPK activation upon metformin treatment despite considerable tumor OCT1 expression, implying that AMPK-independent responses may be integral to the direct anticancer effects of metformin. Indeed, metformin has been shown to suppress mTOR signaling in the absence of AMPK [23].

Additional clinical studies involving metformin treatment of patients with breast cancer have been completed. Consistent with the results of the study described here, a decrease in Ki67 staining was observed in the tumors of patients who received metformin in a randomized window of opportunity study conducted in Scotland [37]. These results differ from those of Bonanni *et al*. [38], who detected no significant effects of metformin on Ki67 but demonstrated a potential association of changes in Ki67 with BMI and HOMA [38]. In addition, metformin did not alter tumor cell proliferation in a recent study completed by Kalinsky *et al*. [39], but patients exhibited reductions in BMI, leptin and cholesterol, indicating systemic effects of metformin. The differences observed in Ki67 staining between these studies could be due to tissue-processing techniques or inherent differences in the patient cohorts (for example, BMI, HOMA), but they could also be the result of differences in the timing of metformin administration before surgery. Nevertheless, the results of the other studies, combined with the changes in cell signaling and receptor expression we observed in the present study, are most consistent with metformin-mediated effects in patients with breast cancer and highlight the potential value of metformin in cancer therapy.

Despite their small cohort sizes, prospectively designed window of opportunity studies provide valuable insight into the mechanism of action of potential anticancer agents and offer an opportunity for the identification of biomarkers of treatment sensitivity and resistance. The present study’s sample size (n = 39) was preplanned and powered to detect a change in Ki67, which was successfully demonstrated previously [24]. Taking into account the potential caveats of small cohort sizes, a limited set of hypotheses directly related to the biological effects of metformin were assessed in an attempt to elucidate its mechanism of antitumor action. The results reported here demonstrate that short-term administration of metformin in patients with breast cancer who do not have diabetes induces tumor-specific changes in IR expression and cell signaling, which are consistent with beneficial anticancer effects of the drug.

## Conclusions

Decreased tumor expression of the IR, combined with reduction in PI3K and Ras-MAPK signaling following metformin administration, points to the indirect insulin-dependent effects of metformin as its mechanism of antitumor action in the neoadjuvant setting. These findings raise the possibility that fasting insulin levels and tumor IR expression may represent biomarkers of metformin sensitivity. Moreover, it is clear that further insight into the impact of AMPK on tumor biology, as well as that of AMPK-independent effects on cell signaling, are needed to develop a comprehensive strategy in modeling possible metformin benefit. Future prospective clinical trials will be integral to the identification of biomarkers that predict metformin benefit and the evaluation of its efficacy as a potential cancer therapy. We are currently investigating some of these concepts in patients enrolled in the NCIC CTG MA.32 study, an ongoing adjuvant trial of 3,649 women with early-stage breast cancer who are receiving metformin versus placebo in addition to standard therapy [40].

## Additional files

Additional file 1:
**“Window of opportunity” clinical trial design.**
Additional file 2:
**Optimization of antibodies against p-Akt (S473) and p-ERK1/2 (T202/Y204) for immunohistochemistry.**
Additional file 3:
**Optimization of antibodies against p-AMPK (T172) and p-ACC (S79) for immunohistochemistry.**
Additional file 4:
**Optimization of the antibody against OCT1 for immunohistochemistry.**
Additional file 5:
**Tumor characteristics table.**
Additional file 6:
**Cytoplasmic and nuclear Allred scores for p-Akt S473.**
Additional file 7:
**Correlative analyses of variables in patients treated with metformin.**
Additional file 8:
**Relationship between reduction in Ki67 and the scaled joint change in serum insulin, tumor IR and p-Akt.**

## Abbreviations

ACC: Acetyl coenzyme A carboxylase
AICAR: 5-aminoimidazole-4-carboxamide-1-β-d-ribofuranoside
AMPK: AMP-activated protein kinase
BMI: Body mass index
DAB: 3,3′-diaminobenzidine
EGF: Epidermal growth factor
ER: Estrogen receptor
ERK: Extracellular signal-regulated kinase
FFPE: Formalin-fixed, paraffin-embedded
Her2: Human epidermal growth factor receptor 2
HOMA: Homeostatic model assessment
IGFR-1: Insulin-like growth factor 1 receptor
IHC: Immunohistochemistry
IQR: Interquartile range
IR: Insulin receptor
LKB1: Liver kinase B1
MAPK: Mitogen-activated protein kinase
MEK1/2: Mitogen-activated protein kinase kinase 1/2
mTOR: Mammalian target of rapamycin
mTORC1: Mammalian target of rapamycin complex 1
OCT1: Organic cation transporter 1
PI3K: Phosphatidylinositol 3-kinase
PKB: Protein kinase B
PR: Progesterone
