# Supplementary material for: Changes in insulin receptor signaling underlie neoadjuvant metformin administration in breast cancer: a prospective window of opportunity neoadjuvant study
Source: Breast Cancer Res. 2015 Mar 3;17(1):32. doi: 10.1186/s13058-015-0540-0 (PMC4381495; doi:10.1186/s13058-015-0540-0)
Supplement: Additional file 1: — “Window of opportunity” clinical trial design. [file 13058_2015_540_MOESM1_ESM.pdf]

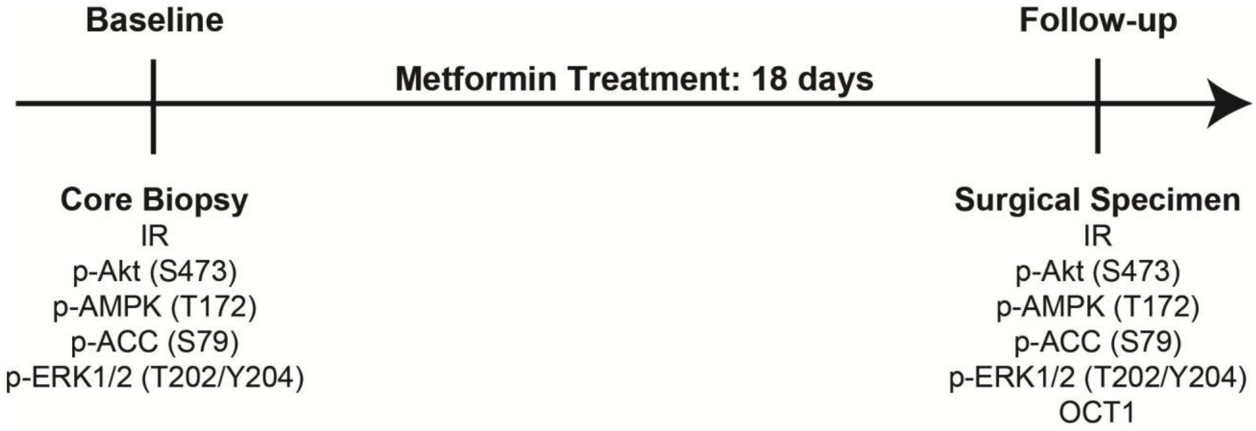

Additional File 1: "Window of opportunity" clinical trial design. Patients received metformin for a median of 18 days. Core biopsies were obtained pre-metformin treatment and specimens were collected after metformin treatment at the time of surgery for analysis of protein expression by immunohistochemistry (IHC). The proteins examined pre- and post-treatment by IHC are listed.
