# Supplementary material for: Changes in insulin receptor signaling underlie neoadjuvant metformin administration in breast cancer: a prospective window of opportunity neoadjuvant study
Source: Breast Cancer Res. 2015 Mar 3;17(1):32. doi: 10.1186/s13058-015-0540-0 (PMC4381495; doi:10.1186/s13058-015-0540-0)
Supplement: Additional file 2: — Optimization of antibodies against p-Akt (S473) and p-ERK1/2 (T202/Y204) for immunohistochemistry. [file 13058_2015_540_MOESM2_ESM.pdf]

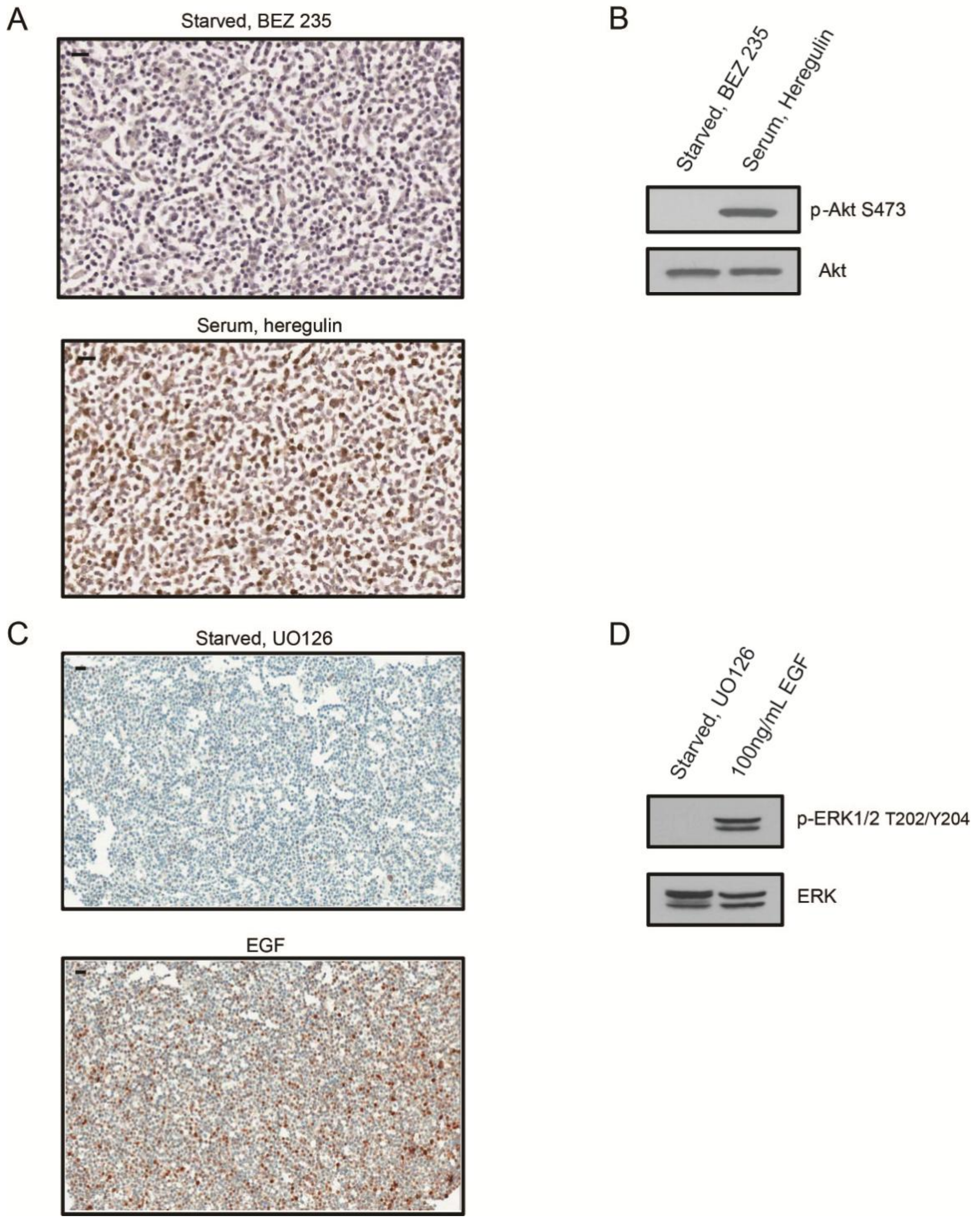

Additional File 2: Optimization of antibodies against p-Akt (S473) and p-ERK1/2 (T202/Y204) for immunohistochemistry. The antibodies for p-Akt (S473) and p-ERK1/2 (T202/Y204) were optimized for IHC using MCF-7 cells. For p-Akt antibody optimization, cells were starved and treated with 200nM BEZ235 for 1 hour or stimulated with serum and 100ng/ml heregulin for 20 minutes. For the p-ERK1/2 antibody, cells were starved of serum overnight and then treated with either 10uM of the MEK1/2 inhibitor UO126 for 1 hour or with media containing 100ng/ml EGF for 10 minutes. p-Akt (S473) and p-ERK1/2(T202/Y204) signal was detected by IHC staining **(A and C)** and confirmed by Western blotting **(B and D)**. Scale bar 30 um (A and C).
