# Supplementary material for: Changes in insulin receptor signaling underlie neoadjuvant metformin administration in breast cancer: a prospective window of opportunity neoadjuvant study
Source: Breast Cancer Res. 2015 Mar 3;17(1):32. doi: 10.1186/s13058-015-0540-0 (PMC4381495; doi:10.1186/s13058-015-0540-0)
Supplement: Additional file 3: — Optimization of antibodies against p-AMPK (T172) and p-ACC (S79) for immunohistochemistry. [file 13058_2015_540_MOESM3_ESM.pdf]

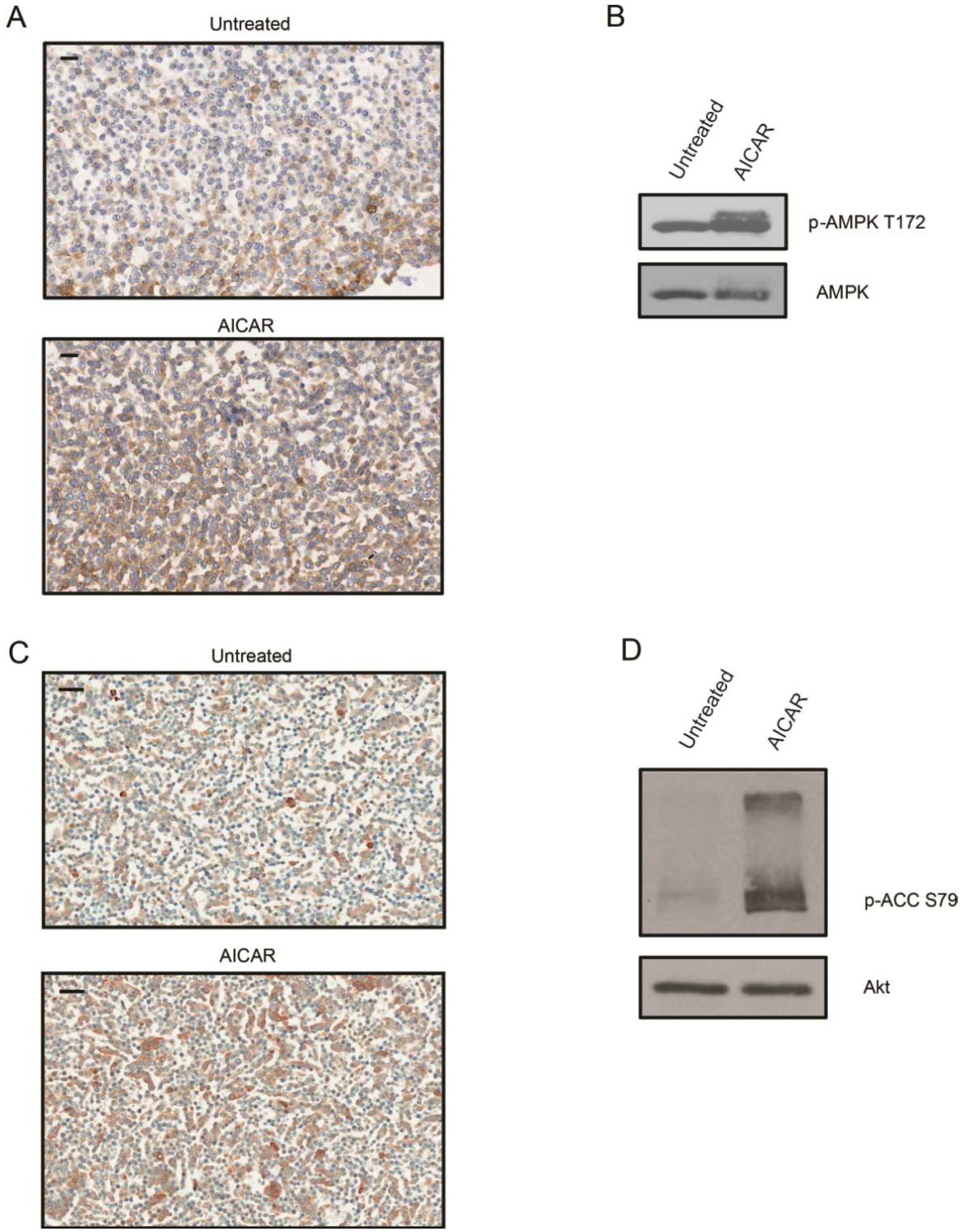

Additional File 3: Optimization of antibodies against p-AMPK (T172) and p-ACC (S79) for immunohistochemistry. The antibodies for p-AMPK (T172) and p-ACC (S79) were optimized for IHC using MCF-7 cells. Cells were left untreated or treated with 2mM AICAR for 3 hours. p-AMPK (T172) and p-ACC (S79) signal was detected by IHC staining **(A** and **C)** and confirmed by Western blotting **(B** and **D)**. Scale bar 30 um (A), 60 um (C).
