# Supplementary material for: Changes in insulin receptor signaling underlie neoadjuvant metformin administration in breast cancer: a prospective window of opportunity neoadjuvant study
Source: Breast Cancer Res. 2015 Mar 3;17(1):32. doi: 10.1186/s13058-015-0540-0 (PMC4381495; doi:10.1186/s13058-015-0540-0)
Supplement: Additional file 4: — Optimization of the antibody against OCT1 for immunohistochemistry. [file 13058_2015_540_MOESM4_ESM.pdf]

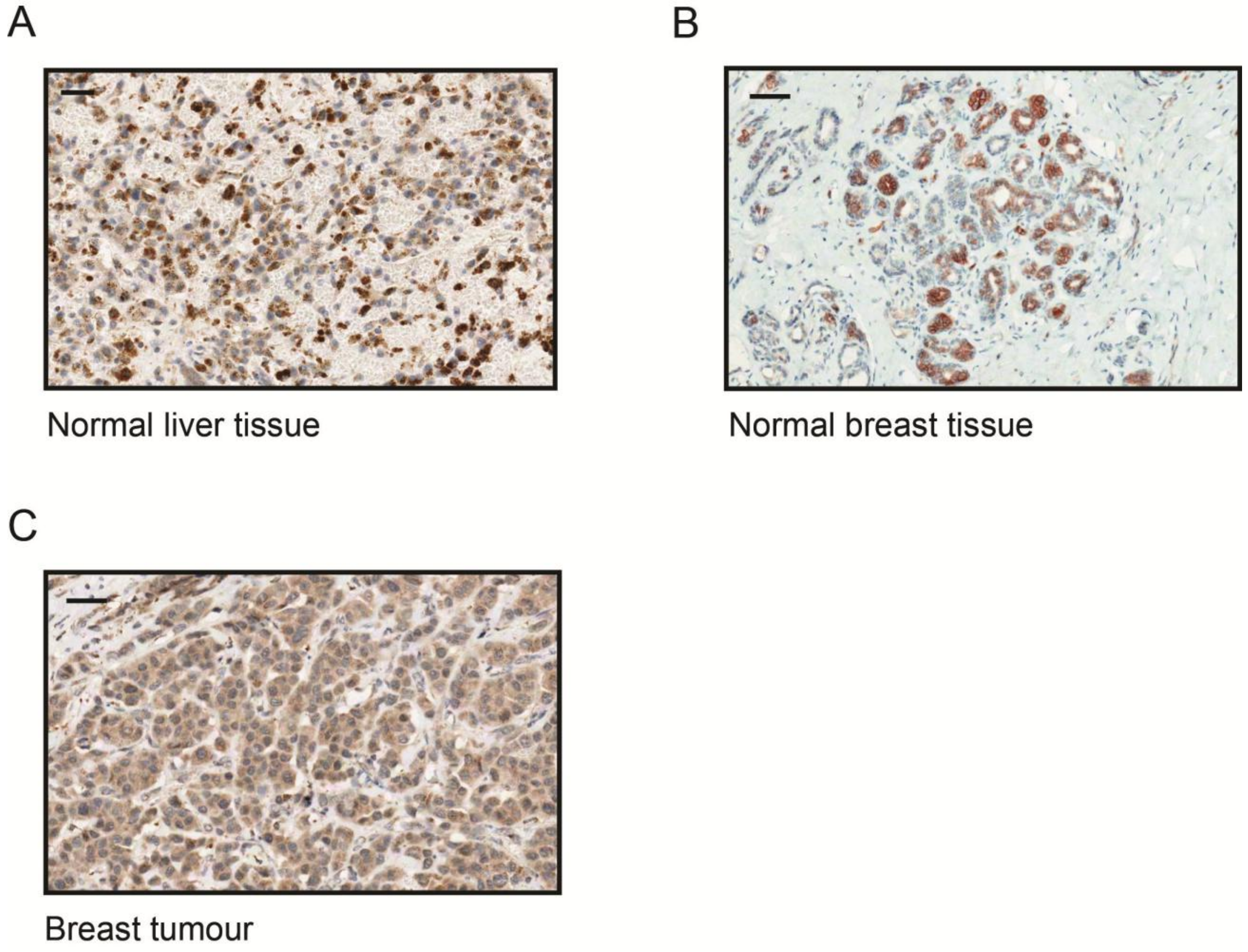

Additional File 4: Optimization of the antibody against OCT1 for immunohistochemistry. The antibody for OCT1 was optimized using formalin fixed-paraffin embedded tissue from normal human liver **(A)** and breast **(B)**, as well as tissue from breast tumours **(C)**. Scale bar 30 um (A and B), 60 um (C).
