# Supplementary material for: Changes in insulin receptor signaling underlie neoadjuvant metformin administration in breast cancer: a prospective window of opportunity neoadjuvant study
Source: Breast Cancer Res. 2015 Mar 3;17(1):32. doi: 10.1186/s13058-015-0540-0 (PMC4381495; doi:10.1186/s13058-015-0540-0)
Supplement: Additional file 5: — Tumor characteristics table. [file 13058_2015_540_MOESM5_ESM.pdf]

Additional file 5: Tumour characteristics

Characteristic	n (%)
Tumour Grade	
Grade I	3 (7.7%)
Grade II	20 (51.3%)
Grade III	16 (41%)
Tumour stage	
T1	20 (51.3%)
T2	16 (41%)
T3	3 (7.7%)
Nodal Status	
Negative	24 (61.5%)
Positive	15 (38.5%)
ER	
Positive	33 (84.6%)
Negative	6 (15.4%)
PgR	
Positive	32 (82.1%)
Negative	7 (17.9%)
HER2	
Positive	5 (12.8%)
Negative	34 (87.2%)
