# Supplementary material for: Changes in insulin receptor signaling underlie neoadjuvant metformin administration in breast cancer: a prospective window of opportunity neoadjuvant study
Source: Breast Cancer Res. 2015 Mar 3;17(1):32. doi: 10.1186/s13058-015-0540-0 (PMC4381495; doi:10.1186/s13058-015-0540-0)
Supplement: Additional file 6: — Cytoplasmic and nuclear Allred scores for p-Akt S473. [file 13058_2015_540_MOESM6_ESM.pdf]

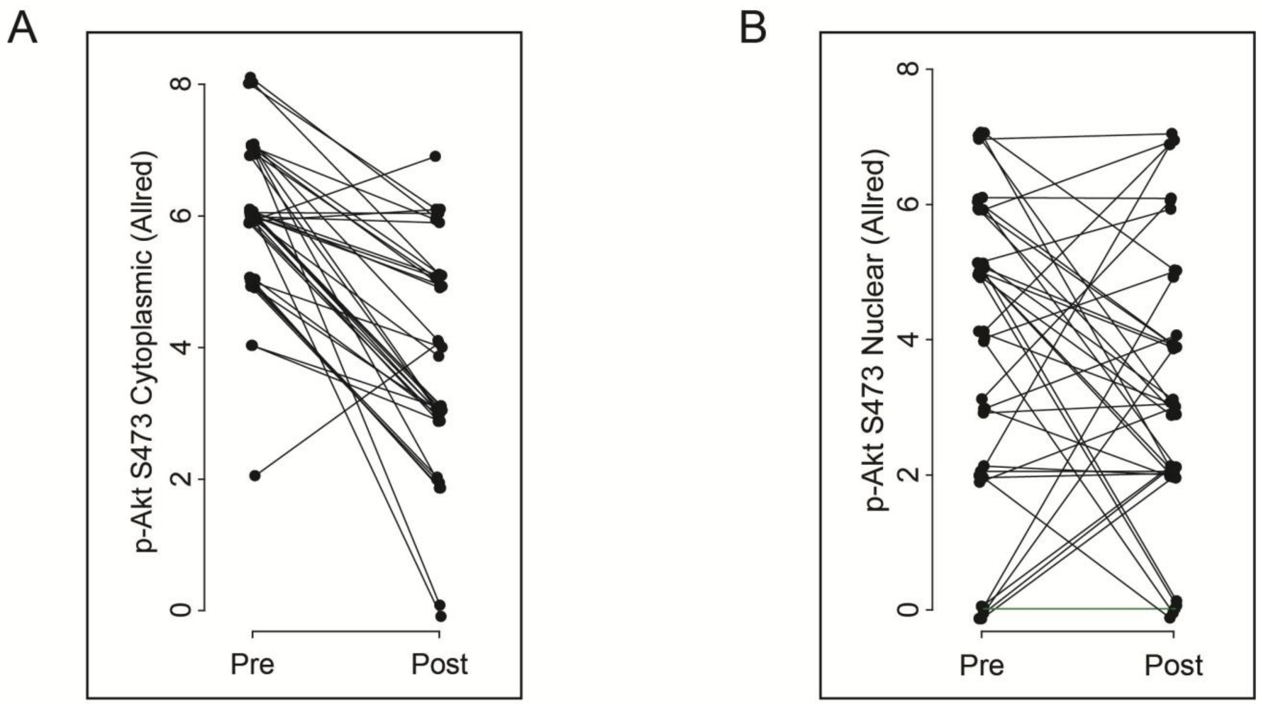

Additional File 6: Cytoplasmic and nuclear Allred scores for p-Akt S473. Allred scores for cytoplasmic **(A)** and nuclear **(B)** p-Akt S473 as measured pre- and post-metformin treatment.
