# Supplementary material for: Changes in insulin receptor signaling underlie neoadjuvant metformin administration in breast cancer: a prospective window of opportunity neoadjuvant study
Source: Breast Cancer Res. 2015 Mar 3;17(1):32. doi: 10.1186/s13058-015-0540-0 (PMC4381495; doi:10.1186/s13058-015-0540-0)
Supplement: Additional file 8: — Relationship between reduction in Ki67 and the scaled joint change in serum insulin, tumor IR and p-Akt. [file 13058_2015_540_MOESM8_ESM.pdf]

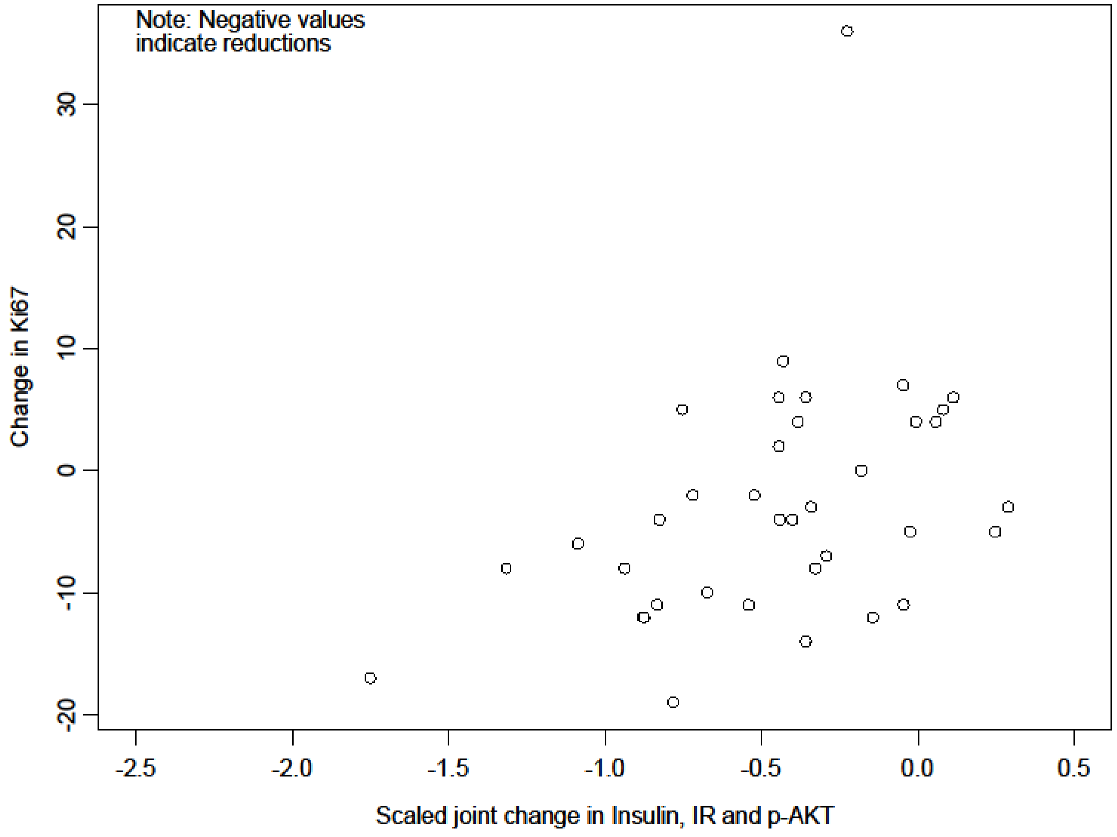

Additional file 8: Relationship between reduction in Ki67 and the scaled joint change in serum insulin, tumour IR, and p-Akt. The joint change was obtained by summing the three variables (insulin, IR, p-Akt) after scaling each one such that its biggest observed reduction became -1. Spearman rank correlation 0.41, p=0.012.
